# Exosomes as mediators of neuroinflammation

**DOI:** 10.1186/1742-2094-11-68

**Published:** 2014-04-03

**Authors:** Archana Gupta, Lynn Pulliam

**Affiliations:** 1 Department of Microbiology and Immunology, Drexel University College of Medicine, 245 North 15th Street, Philadelphia, PA 19102, USA; 2 Departments of Laboratory Medicine and Medicine, San Francisco and Veterans Affairs Medical Center, University of California, 4150 Clement St (113A), San Francisco, CA 94121, USA

**Keywords:** Exosomes, Monocytes, Neuroinflammation, CNS

## Abstract

Exosomes are membrane-bound nanovesicles that are shed by cells of various lineages under normal as well as pathological conditions. Previously thought to be ‘extracellular debris’, exosomes have recently generated immense interest following their discovery as mediators of intercellular communication by delivering functional proteins, mRNA transcripts as well as miRNAs to recipient cells. Although suggested to primarily serve as signaling organelles which also remove unwanted cellular components in the brain, accumulating evidence suggests that exosomes can also significantly contribute to the development of several neuropathologies. Toxic forms of aggregated proteins such as α-synuclein, amyloid β and prions, that are responsible for the development of Parkinson’s disease, Alzheimer’s disease and Creutzfeldt-Jacob disease (CJD) respectively, have been shown to get effectively packaged into exosomes and spread from one cell to another, initiating an inflammatory cascade. In addition, exosomes secreted by resident brain cells in response to pathogenic stimuli such as viral proteins can also influence bystander cells by the transfer of dysregulated miRNAs that suppress the expression of essential genes in the recipient cells. Given the relevance of exosomes in brain communication and neuropathogenesis, novel therapeutic strategies are now being developed that exploit the biology of these vesicles to deliver anti-inflammatory molecules to the CNS. Exosomes may alter the way we think about brain disorders and their treatments.

## Introduction

Disorders of the central nervous system (CNS) can arise from a variety of etiologies including neurodegeneration, autoimmunity and infection. Although several mechanisms have been implicated, one common player capable of mediating the pathogenesis of these diseases has recently emerged. Exosomes are spherical, membrane-bound nanovesicles that are shed by a majority of cell types in the body. They are released under normal as well as pathological conditions and mediate intercellular communication between cells of various lineages. Initially thought to be waste bags that carried unwanted proteins shed by cells, exosomes were only recently discovered to contain not only cellular proteins but also mRNA transcripts as well as miRNA from the host cell. Importantly, exosomes can to be internalized by cells of various lineages and functionally alter the physiological environment of the recipient cell. These observations quickly changed the perception of exosomes from organelles carrying cell debris to messengers for cell communication. The past few years have witnessed a deluge of information in the field of extracellular vesicles and their implications to not only serve as potential biomarkers of several diseases, but also as a mechanism for the development of many pathological conditions. The information unraveled thus far is just the tip of the iceberg. The discovery of exosomes as extracellular vesicles packed with functional cellular cargo bears the potential to significantly influence the future of diagnostics and drug delivery. This review focuses on the current knowledge of exosomes in the pathogenesis of various CNS disorders, their potential to serve as therapeutic agents to treat neuroinflammation as well as biomarkers to diagnose various brain diseases.

### Exosome biogenesis

**Figure 1:**
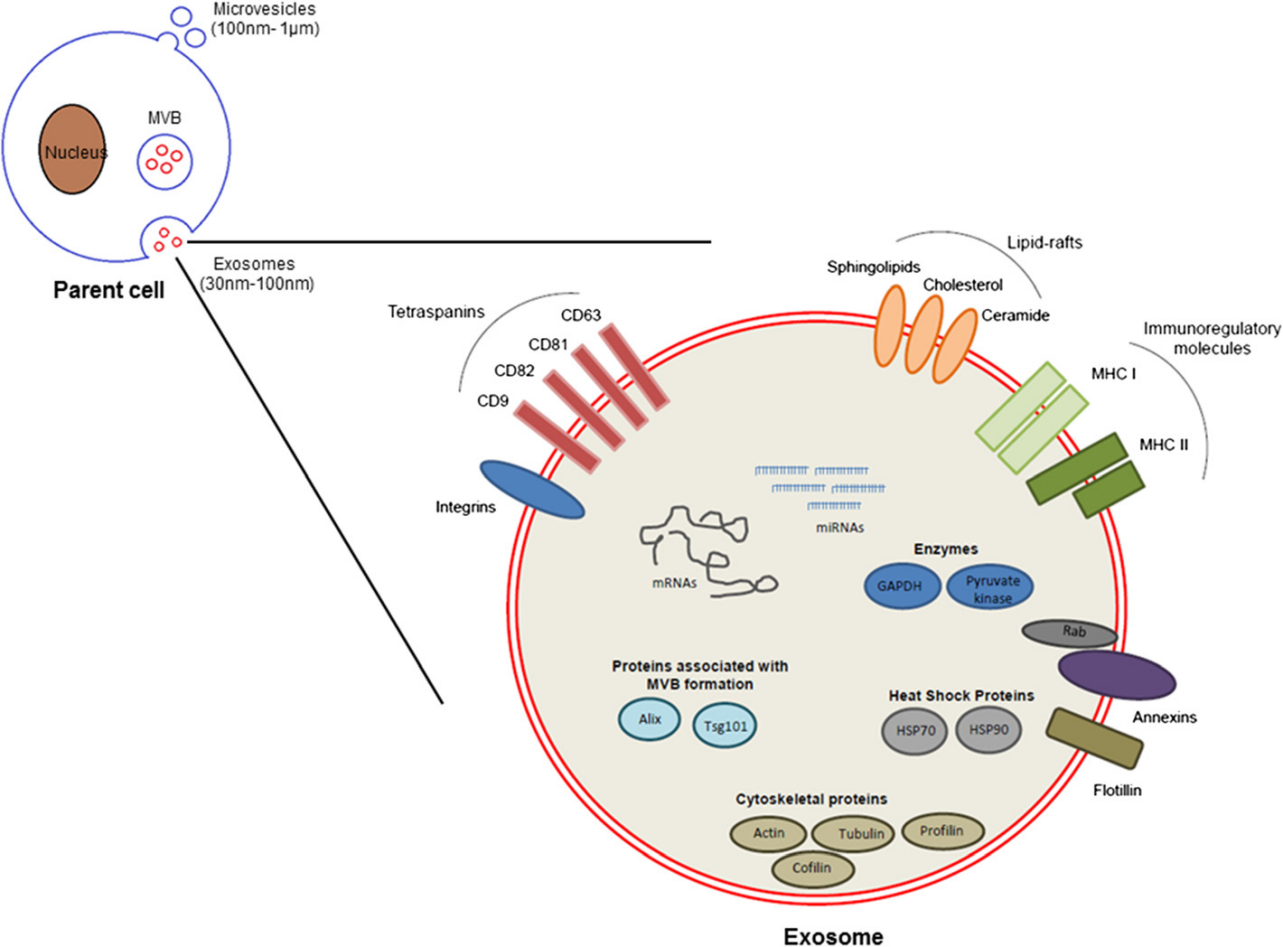
**Composition of exosomes.** Exosomes and microvesicles are membrane-bound extracellular vesicles secreted from various cell types. Exosomes are 30 to 100 nm in size and packed with a variety of cellular components including mRNAs, miRNAs and proteins that are often selectively packaged from the cell the vesicles are shed. The exosome membrane is decorated with various proteins involved in antigen presentation called major histocompatibility complexes (MHC I and II), targeting and adhesion (integrins and tetraspanins), membrane trafficking (annexins and Rab proteins) as well as lipid-rafts.

Exosomes are 30 to 100 nm spherical vesicles that are released in to the extracellular space by diverse cell types and mediate a variety of cellular functions [1,2]. Similar to microvesicles, exosomes also transport a host of biomolecules to cells of different lineages [3-7]. Together, they are often referred to as extracellular vesicles and can be distinguished not only by their size, but also by the source of their origin. In contrast to microvesicles that are shed by the plasma membrane [7,8], exosomes are formed by the inward budding of the limiting membrane of the late endosome, and encapsulate cellular proteins, RNA as well as miRNAs from the cytoplasm in a non-random fashion [9,10]. Late endosomes that are packed with several such small vesicles (or intraluminal vesicles) are often termed multivesicular bodies (MVBs). The late endosomes are destined to either fuse with the lysosome which leads to the degradation of the contents of the vesicles, or the plasma membrane which allows for the exosomes to be released into the extracellular space. The formation of the intraluminal vesicles is a regulated process that involves the organization of the endosomal membrane into specialized domains that are highly enriched for a specific class of membrane proteins called tetraspanins (Figure 1). These units, also called Tetraspanin-Enriched Membrane Domains (TEMs) recruit proteins required to facilitate vesicular fusion and/or fission [11]. Additionally, the TEMs also recruit potential ligands for receptor-mediated internalization of the exosome by the recipient cell [12]. Several members of the tetraspanin family including CD9, CD63 and CD81 are highly enriched on exosomal membranes and not surprisingly, serve as marker proteins for the vesicles. A second set of proteins involved in the formation of the exosomes are the components of the ESCRT machinery (Endosomal Sorting Complex Required for Transport). Membrane proteins that are signaling-induced ubiquitinated and internalized into early endosomes trigger the recruitment of the ESCRT complexes. ESCRT complexes 0, I, and II directly bind to ubiquitin, and therefore, allow only those proteins that are ubiquitinated to be loaded onto MVBs. However, there are a few exceptions in which proteins that are not ubiquitinated are also sorted into these vesicles; in such instances, sorting is believed to be a passenger effect [13].

In addition, ESCRT I, and II are suggested to drive the budding of the intraluminal vesicles (ILV), while ESCRT III is required for the cleavage of the bud to form the ILV. Another important protein, Alix, that is also often used as an exosomal marker and interacts with TSG101 and CHMP4, participates in several processes of the exosome biogenesis including cargo packaging and vesicle formation.

### Composition of exosomes

Exosomes are lipid-bilayer enclosed vesicles that carry cellular proteins, mRNA as well as miRNAs. The membrane proteins appear in the same orientation as on the cell membrane, owing to two invaginations, one at the surface of the plasma membrane, during the formation of the endosome, and the second by the inward budding of the endosomal membrane. Considering that the formation of exosomes is an active process, there are several membrane proteins and lipids that are unique to these vesicles compared to the cell surface. Owing to their endosomal origin, exosomes harbor tetraspanins (CD9, CD63, CD81 and CD82), proteins required for membrane transport and fusion (annexins, Rab proteins, flotillin), proteins associated with MVB biogenesis (Alix, TSG101), and heat shock proteins (Hsc70, Hsp90) as previously reviewed [8,14] (Figure 1). The vesicles also carry a variety of cytoskeletal proteins (actin, tubulin, profilin, cofilin, and so on) and metabolic enzymes (GAPDH and pyruvate kinase). Additionally, the exosomal membrane is enriched with lipid-rafts including cholesterol, sphingolipids, ceramide and glycerophospholipids containing long and saturated fatty-acyl chains. Interestingly, exosomes secreted from antigen-presenting cells such as dendritic cells also exhibit functional major histocompatibility complexes (MHC I and II) on their surface [15,16].

The cell type the exosome is derived from heavily influences the protein content of the exosomes. Several proteomic studies performed on exosomes isolated from cancerous cells reveal the presence of various factors associated with tumor progression such as metastasis, angiogenesis and signal transduction [17]. The exosomal pathway can also be hijacked by viruses to spread viral proteins to other cell types, without actually infecting them, and dysregulating their function. For instance, CD4+ T cells infected by HIV release the viral protein Nef in exosomes which induces apoptosis of bystander T cells [18].

Exosomes are highly enriched in small RNAs. A bioanalyzer profile of exosomal RNA indicates that the predominant RNA species in these vesicles ranges from 4 to 40 nucleotides in length [19], consistent with miRNAs. An increasing body of evidence suggests that exosomal miRNA is functional in the recipient cell and can repress the expression of the target genes in the latter [4,20-22]. The ability of such miRNAs to exert a biological effect indicates that they are stable as well as associated with cellular proteins that render them functional. Several studies show that exosomal miRNAs are in fact associated with Ago2 proteins, albeit to various degrees [23]. MVBs are proposed to be a site for the congregation of miRNA pathway components, mature miRNAs as well as the target transcripts [24], and may therefore be the route for the packaging of these elements into exosomes. Apart from cellular miRNAs, specific virus-encoded miRNAs can also be packaged into exosomes of infected cells. Epstein-Barr virus (EBV) has been shown to encode viral miRNAs BHRF1 and BART that can be shed in nanovesicles from infected B-cells and suppress the immunoregulatory gene CXCL11 in non-infected neighboring monocyte-derived dendritic cells [25]. In addition, HIV-encoded trans-activation response element (TAR)-miRNA can be released by infected cells in nanovesicles, influencing the susceptibility to infection of cells that internalize it [26]. Clearly, viruses have evolved strategies to usurp the host exosomal machinery in order to propagate and attenuate host antiviral response.

mRNA transcripts, also enclosed in exosomes, can be readily translated to proteins by the cell receiving the vesicles. Valadi *et al*. [27] were among the first to demonstrate that exosomal mRNA from mouse cells could be translated into murine proteins by human mast cells. However, the majority of these transcripts appear to be highly enriched in the 3′-untranslated regions, suggesting that the mRNA in these vesicles may play more of a regulatory rather than a functional role [28].

Uptake of exosomes is an energy-dependent active process [4,29]. Various proteins and lipids present on the exosomal membrane such as tetraspanins and phosphatidylserine take part in receptor-mediated endocytosis by interacting with their complementary molecules present on the plasma membrane of the target cell [29,30] and initiate internalization. The endocytic pathway involved in exosomal uptake however varies from cell-to-cell. Although phagocytosis is the major pathway for exosome internalization by professional phagocytic cells such as liver Kupffer cells and dendritic cells [30,31], uptake of vesicles by macropinocytosis has also been demonstrated for the brain resident macrophages, microglia [32]. In contrast, non-phagocytic cells employ both clathrin-dependent [29] and independent endocytic pathways [33] to internalize exosomes.

### Exosomes in CNS communication

Most cells in the CNS, including neurons, astrocytes, oligodendrocytes and microglia shed exosomes. These extracellular vesicles are secreted by neural cells under both normal and pathological conditions and have been isolated not just from the cerebrospinal fluid [34] but also from adult human brain [35]. The suggested roles of exosomes in CNS are to get rid of waste membrane and cellular materials, and to serve as messengers for communication between neural cells. In that regard, exosomes can participate not only in the development of the CNS, but also in regulating synaptic activity as well as regeneration following injury. For instance, maturing neurons regulate oligodendrocyte differentiation by exerting an effect on the release of oligodendrocyte-derived autoinhibitory exosomes [36]. In contrast, exosomes released from oligodendrocytes in response to glutamate activation can influence neuronal metabolism and exhibit neuroprotective function by the functional transfer of oligodendrocyte cargo [37].

Synapses are essential to the function of neurons. They enable signal transduction from one neuron to the next by the transfer of chemical messages, inducing changes in second messengers that trigger a response leading to transmission of the signal. Exosomes shed by different neural cells can participate in synaptic excitation by transferring essential proteins for neurotransmission to neurons. Synapsin, a synaptic vesicle-associated protein implicated in neural development was shown to be released by glial cells in exosomes under conditions of high neuronal activity or cell stress [38]. These exosomes increase neuronal survival and promote neurite growth under adverse environmental conditions. Microglial cells can also stimulate synaptic activity in neurons via exosomes [39]. Microglial exosomes enhance ceramide and sphingosine metabolism in the recipient neurons resulting in an increase in neurotransmission.

**Figure 2:**
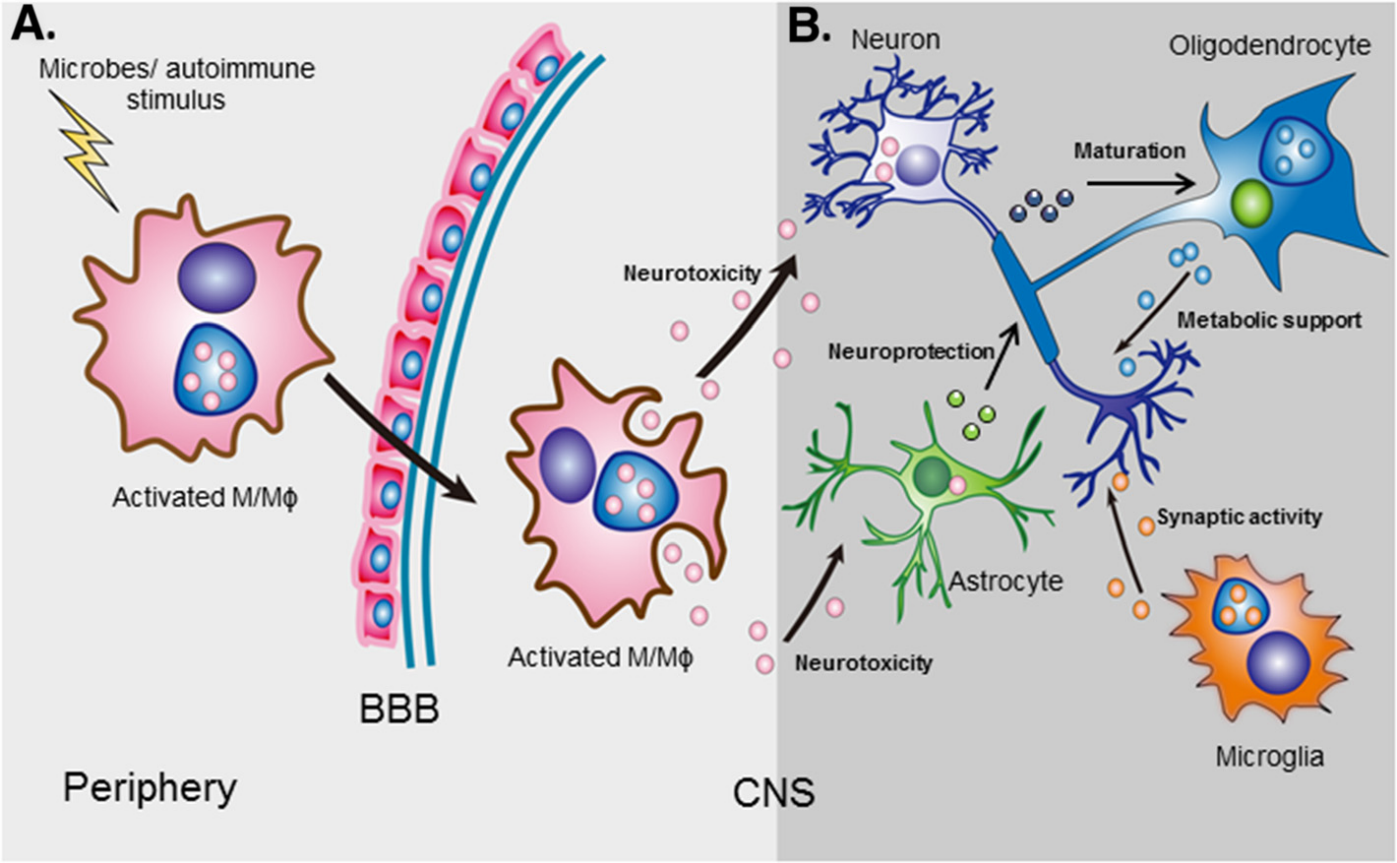
**Exosomal cross talk from the periphery and within the central nervous system (CNS). (A)** Influence of peripheral inflammation. Inflammation in the periphery can lead to neural cell impairment by virtue of activated monocyte-derived exosomes. Activated monocyte/macrophages traffic across the blood-brain barrier (BBB) at a high frequency and shed exosomes that can harbor pathogenic cellular products including dysregulated miRNAs, mRNAs and proteins as well as microbial by-products. Once internalized by neural cells, exosomes can functionally transfer the neurotoxic cargo to astrocytes and neurons causing neurodysfunction. **(B)** Normal intercellular communication in the brain mediated by exosomes. The release and uptake of exosomes by various cell types in the brain is part of normal brain communication. Astrocytes release exosomes enriched in heat shock proteins that serve a neuroprotective function during stress conditions. Exosomes shed by microglia, on the other hand, promote neurotransmission by stimulating ceramide and sphingosine syntheses. Oligodendrocyte-derived exosomes offer metabolic support to neurons in response to glutamate activation while neuronal exosomes regulate differentiation of the former.

Exosomes can mediate neuroprotection. Astrocytes, known primarily to provide nutritional support to neurons, secrete elevated levels of heat shock protein 70 (Hsp70) in response to oxidative stress and hyperthermia [40] by increasing the survivability of neighboring neurons during injury [41]. In the peripheral nervous system, Schwann cells communicate with and support axonal regeneration after nerve damage through exosomes. The exosomes mediate such effect, at least in part by the suppression of the activity of RhoA, a GTPase activated in response to injury and inhibiting axonal regeneration at growth cones [42] (Figure 2).

### Exosomes and neuroinflammatory disorders

While exosomes mediate several vital processes required for normal brain functioning [37,39,42], they are also involved in the pathogenesis of many neuroinflammatory disorders, both infectious [20,43] and neurodegenerative in nature [44-46]. Increasing evidence suggests that exosomes serve as carriers to misfolded and pathogenic proteins [43,47], initiating or propagating the disease in neighboring cells. In addition to cytoplasmic proteins, aberrantly expressed cellular miRNAs, selectively packaged and transported in exosomes to neural cells [19,35], can further dysregulate gene expression of the recipient cell.

#### Exosomes in the pathogenesis of infectious CNS diseases

##### 

**HIV-associated neurocognitive disorders (HAND)** HIV infection is associated with an elevated risk for neurological impairment. Abuse of drugs such as opiates during infection further increases the incidence and progression of HIV-associated neurodysfunction, although the mechanism for the synergistic effect remains unknown. A recent study by Hu *et al*. [20] suggested that co-exposure of astrocytes to morphine and HIV Tat protein induces the release of exosomes enriched in miR-29b which can impair neuronal function. The mechanism involves the functional transfer of miR-29b to neurons leading to the suppression of the neuroprotective protein platelet-derived growth factor (PDGF)-B expression. The expression of PDGF is also suppressed, and coupled to elevated levels of miR-29b, in the basal ganglia of the brains of simian immunodeficiency virus-infected macaques dependent on morphine, underscoring the possibility of exosomes mediating the neuropathology.

HIV-infected cells can also release the TAR RNA packaged into exosomes [26]. These exosomes are proposed to increase the susceptibility of the recipient cells, such as astrocytes, to HIV infection by the interference of host gene expression caused by 3′TAR-miRNA.

##### 

**Prion disease** Prion diseases are infectious neurodegenerative disorders associated with the accumulation of an abnormally folded isoform of the prion protein (PrP), scrapie (PrPSc), considered as the infectious agent. The misfolded protein can induce conformational conversion of cellular prion protein into its pathogenic isoform by a protein-only template-directed mechanism. The disease, often fatal in nature includes CJD in humans, scrapie in sheep, and bovine spongiform encephalopathy in cows. It is characterized by massive neuronal loss leading to the formation of large spongiform vacuoles in the brain.

Although conditioned media from prion-infected neurons can spread infection to uninfected cells, the mechanism for such dissemination remained elusive for many years. A pioneering study by Fevrier *et al*. [43] demonstrated that PsPSc associates with neuronal exosomes, thereby bypassing the need for cell-to-cell contact. Interestingly, these exosomes were shown to infect not only uninfected recipient cells but also non-neuronal cells [47]. Exosome-associated PrP is processed by a distinct pathway that involves N-terminal modification of the protein and selective loading of PrP glycoforms for incorporation into the vesicles [47].

Another significant observation in regard to the role of exosomes in the pathogenesis of prion disease was made by Bellingham and colleagues [19]. They demonstrated that exosomes secreted by prion-infected neuronal cells have significantly altered miRNA content including elevated expression of several miRNAs associated with neurological disorders such as miRs-29b, 128a and 146a. Alteration of exosomal miRNA content can influence the functioning of the recipient cell by dysregulating the expression of potentially important genes.

#### Exosomes and neurodegeneration

##### 

**Alzheimer’s disease (AD)** AD is the most common form of age-associated dementia in humans [48]. It is characterized by accumulation of extracellular amyloid β (Aβ) plaques and intracellular neurofibrillary tangles of hyperphosphorylated tau protein that ultimately leads to slow and progressive loss of neurons. The Aβ peptide is formed by the sequential proteolytic cleavage of the transmembrane amyloid precursor protein (APP) by two membrane-bound enzymes β-secretase and γ-secretase. Although several different species of Aβ peptides are generated, the longer forms are particularly hydrophobic and prone to aggregation in extracellular Aβ plaques [49]. The tau protein, on the other hand, is a microtubule-stabilizing protein that is predominantly expressed in neurons and regulates axonal transport. Hyperphosphorylation of tau leads to its dissociation from microtubules and aggregation into insoluble fibers in neurons.

The roles of exosomes in mediating the pathogenesis of AD were initially associated with the nanovesicles serving as vehicles for the transport of the Aβ peptides to the extracellular environment. A seminal study by Rajendran *et al*. [44] demonstrated that the cleavage of APP by β secretase occurs in a specific subset of endosomes and is trafficked into MVBs from where it is released in association with exosomes. Interestingly, exosomal proteins such as Alix and flotillin-1 are found to accumulate around amyloid plaques in the brains of AD patients further underscoring the relevance of exosomes in spreading Aβ [44]. Recently, amyloid peptides were shown to induce apoptosis in astrocytes by up-regulating the intracellular expression of the proapoptotic proteins, prostate apoptosis response 4 (PAR4) and ceramide [45]. Importantly, astrocytes exposed to the Aβ peptides could induce apoptosis in bystander astrocytes by the release of exosomes enriched in PAR4 and ceramide, suggesting that the vesicles could either functionally transfer the proteins to the recipient astrocytes or trigger a cascade of events ultimately leading to cell death. In addition to Aβ peptides, exosomes also carry phosphorylated tau protein associated with neurodegeneration [50] and may be responsible for cell-to-cell spread of tau as has been reported previously [51]. Exosome-associated phosphotau is readily detected at elevated levels in the cerebrospinal fluid (CSF) samples of early AD patients and decreases as the disease progresses despite the increase in the overall level of the protein, possibly due to dissociation of tau from the exosome fraction.

##### 

**Parkinson’s disease** After AD, Parkinson’s disease (PD) is the most common neurodegenerative disorder affecting nearly 1% of the population over 50 years old [52]. Patients suffering from PD typically demonstrate movement-related disorders such as resting tremor, and muscle rigidity and cognitive malfunction as the disease progresses. Elevated intracellular levels of aberrant conformations of α-synuclein (α-Syn), a presynaptic neuronal protein, areAccepted frequently associated with PD pathology. Similar to AD, exosomes have been shown to be involved in transporting α-Syn to the extracellular environment and spreading the toxic oligomers to naïve neurons [53]. Uptake of exosomes loaded with α-Syn has been shown to induce cell death in healthy neurons [46] further highlighting the potential of exosomes in the pathogenesis of neurodegenerative disorders. In addition to neurons, other cell types including astrocytes [54] have also been shown to internalize α-Syn deposits released by pathologic neurons. The phenomenon is associated with the formation of inclusion bodies in the latter, as observed in the brains of PD patients [54] as well as initiation of an inflammatory response. Another interesting perspective to the role of exosomes in PD pathogenesis comes from the reports that mutations in several genes involved in the endocytic pathway such as leucine-rich receptor kinase 2 (LRRK2), and vacuolar sorting protein 35 (VPS35), are linked to PD [52]. LRRK2, for example, has been shown to regulate synaptic transmission and its overexpression is associated with suppression in synaptic vesicle endocytosis and exocytosis in neurons [55]. Additionally, a mutation in LRRK2 leads to an abnormal increase in the number of morphologically distinct MVBs [56]. Formation of a large number of MVBs could potentially lead to the accumulation of exosomes enriched in the toxic form of α-Syn and spread the disease to neighboring cells upon release.

### Impact of peripheral inflammation on the CNS via exosomes

Often, conditions associated with inflammation in the periphery impact brain function. For instance, untreated HIV infection is associated with neurocognitive impairment in over 50% of infected subjects [57,58]. Coinfection with hepatitis C virus (HCV) worsens neurological functioning regardless of HIV infection status [59-61]. Subjects with other inflammatory diseases such as systemic lupus erythematosus (SLE) [62] and rheumatoid arthritis (RA) [63] have also been shown to exhibit a high frequency of neurological disturbances. Interestingly, all the above stated disorders are associated with a peripheral type I IFN profile. While no direct correlative analysis has been performed to assess the relationship between peripheral activation and neurodysfunction in SLE and RA despite the high frequency of occurrence, our lab recently showed that the activation state of the circulating monocytes in HIV/HCV coinfection correlates with worsening neurocognitive symptoms [62,63,56].

Circulating monocytes traffic across the blood-brain barrier (BBB) as part of a normal physiological process to replenish the perivascular macrophages. However, during specific inflammatory conditions, a subset of activated monocytes migrate across the BBB at a much greater frequency and mediate events that ultimately lead to neural cell dysfunction [64]. In the context of untreated HIV infection, such transmigration is widely associated with spread of infection to the CNS, and release of neurotoxic pro-inflammatory cytokines from activated monocytes [65]. However, in the post-antiretroviral therapy (ART) era, neurocognitive impairment continues to persist and may be attributed to other mechanisms beside active viral infection [66-71]. The incidence of impairment is worsened during coinfection with HCV, a classical hepatotropic virus. While some studies have suggested that HIV can establish a silent reservoir in the monocytes despite successful viremic control, virus can be intermittently activated and contribute to HAND [65,72,73]. The occurrence of neurocognitive difficulties in certain non-infectious, inflammatory autoimmune disorders affecting the periphery however suggests that perhaps other mechanism (s) may also contribute to brain dysfunction (Figure 2).

Primary human monocytes have been shown to shed exosomes *in vitro* as well as *in vivo*[5,21]. In fact, the majority of the extracellular vesicles found to circulate in the body are shed by platelets and mononuclear cells [5]. Inflammatory signals from the environment can trigger a change in the gene expression and miRNA profile of a cell as well as in the exosomes shed by it. Given that cells release exosomes under both physiological as well as pathological conditions, monocyte-derived vesicles could serve as a vehicle to transport neurotoxic cargo to neural cells or alternatively trigger several signaling pathways by virtue of ligands and receptors on the exosomal surface [74]. Dysregulated miRNAs, or mRNA transcripts packaged into exosomes could be functionally delivered to neural cells and disrupt an array of pathways within the cell, ultimately causing disturbances in brain function. In addition, microbes that infect and replicate in monocyte/macrophages, for instance HIV, may release viral proteins or genomic products in exosomes and elicit an inflammatory response in recipient neural cells. This phenomenon has been shown to occur in T cells infected with HIV [18,26] as well as HCV-infected hepatoma cells [75].

While monocyte/macrophages-derived exosomes can function as a ‘Trojan horse’, exosomes in circulation can also migrate across the BBB and exert a biological effect on neural cells by internalizing the vesicles and releasing exosomes in the CNS, as suggested by Alvarez-Erviti *et al*. [76].

### Exosomes for treating neuroinflammatory disorders

One of the major hurdles in treating neuroinflammatory disorders has been the lack of an optimized delivery strategy that allows the drug to cross the BBB. Exosomes have the potential to serve as the ‘ideal drug delivery vehicle’ owing to their desirable properties such as low immunogenicity, ability to effectively transport a range of biomolecules and interact with a host of target cells, and importantly, amenability to manipulation for personalized medicine.

The concept that exosomes can be packaged with therapeutic agents and used for treating inflammatory conditions *in vivo* was first demonstrated by Sun *et al*. [77]. The study showed that exosomes loaded with curcumin, an anti-inflammatory naturally-occurring polyphenol, not only increased the bioavailability and stability of the compound *in vivo*, but also significantly improved survival in lipopolysaccharide (LPS)-induced septicemia. Building on this finding, Zhang *et al*. [78] later showed that LPS-induced brain inflammation in mice could be alleviated by intranasal administration of curcumin-loaded exosomes. The exosomes were selectively internalized by microglial cells and subsequently induced apoptosis in the activated recipient cells.

Exosomes can be manipulated *ex vivo* to carry not just therapeutic drugs but also short interfering RNAs (siRNAs) targeted against specific genes in the brain [79]. siRNAs are small non-coding RNA sequences that suppress gene expression by degrading the complementary mRNA transcript. Alvarez-Erviti *et al*. [76] demonstrated that exosomes from self-derived dendritic cells suppressed target genes in the brain by the delivery of siRNA to neurons, microglia and oligodendrocytes. The exosomes were targeted specifically to neural cells by transfection of dendritic cells with Lamp2b, an exosomal membrane protein fused to a rabies glycoprotein peptide. The authors also showed that exosomes could be loaded directly with siRNAs by electroporation and gene expression could be silenced in the CNS by intravenous injection of the vesicles. The latter observation suggests that exosomes in the periphery can cross the BBB and regulate brain function. Thus, exosomes have the potential to serve as a non-invasive intervention for the successful delivery of therapeutic agents to the brain.

Although the proof-of-concept studies are very encouraging, we are only beginning to understand the potential of exosomes as a viable therapeutic intervention for several hard-to-treat medical conditions. A few key considerations for the development of exosome-based therapies to treat neurological disorders are likely to revolve around: 1) specificity to achieve targeted delivery, 2) bioavailability and half-life of the exosomes *in vivo* in order to determine frequency of administration, 3) site of exosome administration and 4) potential toxicity associated with non-targeted effects.

### Exosomes as biomarkers for neuroinflammation

Changes in the cellular environment often impact the content of exosomes shed by the cell. This phenomenon has the potential to extend the application of these nanovesicles as biomarkers for various pathological conditions wherein specific proteins/mRNAs and/or miRNAs that are altered in a diseased state can be detected in exosomes for early diagnosis. Indeed, Banigan *et al*. [35] recently showed that exosomal miRNAs recovered from postmortem prefrontal cortices of subjects diagnosed with schizophrenia and bipolar disorders were different from matched controls. Brain tissue samples from schizophrenic patients had significantly elevated levels of exosomal miR-497 while those from bipolar disorder had upregulation of miR-29c. Future studies focused on identifying disease-associated exosomal miRNAs in the CSF of living patients will perhaps validate the utility of exosomes to serve as biomarkers in schizophrenia and bipolar disorder. Apart from exosomal miRNAs, mRNA transcripts differentially packaged into exosomes released from diseased cells have also been proposed as biomarker candidates. Skog *et al*. [80] demonstrated that subjects with glioblastoma had detectable levels of vesicle-associated epidermal growth factor receptor (EGFRvIII) transcript in the serum that could be amplified by RT-PCR. No amplification of the transcript was detected in vesicles isolated from healthy subjects. Interestingly, this variant of EGFR is specific to tumors and its specific detection only in vesicles isolated from cancer patients makes this transcript a good biomarker candidate.

## Conclusions

The relevance of exosomes in the pathogenesis of several CNS disorders has only begun to be explored. Given their ability to mediate intercellular communication between cells, it is not surprising that exosomes represent one of the key players in transporting neurotoxic cargo and disseminating disease in the brain. Nevertheless, the potential of exosomes to be manipulated for the transport of therapeutic agents has generated heightened interest in the field, as these vesicles are capable of circumventing the major hurdles that are associated with the delivery of drugs across the BBB.

## Abbreviations

Aβ: amyloid β; AD: Alzheimer’s disease; APP: amyloid precursor protein; ART: anti-retroviral therapy; BBB: blood-brain barrier; CJD: Creutzfeldt-Jacob disease; CNS: central nervous system; CSF: cerebrospinal fluid; EBV: Epstein-Barr virus; EGFR: epidermal growth factor receptor; ESCRT: Endosomal Sorting Complex Required for Transport; HAND: HIV-associated neurocognitive disorders; HCV: hepatitis C virus; HIV: human immunodeficiency virus; HSP7: heat shock protein 70; IFN: interferon; ILV: intraluminal vesicles; LPS: lipopolysaccharide; LRRK2: leucin-rich receptor kinase 2; MHC: major histocompatibility complex; miRNAs: microRNAs; MVB: multivesicular bodies; PAR4: prostate apoptosis response 4; PD: Parkinson’s disease; PDGF: stands for platelet-derived growth factor; PrP: prion protein; PrPSc: prion protein scrapie isoform; RA: rheumatoid arthritis; RT-PCR: real time-polymerase chain reaction; siRNAs: short interfering RNAs; SLE: systemic lupus erythematosus; α-Syn: α-synuclein; TAR: trans-activation response element; TEM: Tetraspanin-Enriched Membrane domains; VPS35: vacuolar sorting protein 35

## Competing interests

The authors declare that they have no competing interests.

## Authors’ contributions

Both AG and LP contributed to the writing of this manuscript. Both authors read and approved the final manuscript.
